# An Analysis of Oxidative Changes and the Fatty Acid Profile in Stored Poultry Sausages with Liquid and Microencapsulated Fish Oil Additives

**DOI:** 10.3390/molecules26144293

**Published:** 2021-07-15

**Authors:** Krzysztof Kawecki, Jerzy Stangierski, Piotr Konieczny

**Affiliations:** Department of Food Quality and Safety Management, Faculty of Food Science and Nutrition, Poznan University of Life Sciences, Wojska Polskiego 31/33, 60-624 Poznan, Poland; piotr.konieczny@up.poznan.pl

**Keywords:** chicken sausages, fish oil, microencapsulated fish oil, lipid oxidation, fatty acid profile, vacuum-packing, modified-atmosphere-packing, sensory analysis, in vitro digestion

## Abstract

This study deals with the fatty acid profile and oxidative changes (TBARS) in vacuum-packed (VP) or modified-atmosphere-packed (MAP) finely-comminuted poultry sausages with liquid fish oil and microencapsulated fish oil (MC) additives. An analysis of omega-3 fatty acids (EPA and DHA) showed that their content in the samples with the fish oil additive decreased from the initial value of 0.22 g∙100 g^−1^ of the product to 0.18 g∙100 g^−1^ (MAP) and 0.17 g∙100 g^−1^ (VP), respectively. After in vitro digestion, the total EPA and DHA content in the sample with microencapsulated oil amounted to 0.17 g∙100 g^−1^ of the product. The TBARS values showed the VP samples with both forms of the fish oil additive had the lowest values on the first day of storage. Storage of the samples for 21 days caused a slight increase in the degree of lipid oxidation. The research indicated that the forms of the oil additive did not have a negative influence on the sensory features or the physicochemical properties of the sausages. The EPA and DHA levels in samples with liquid fish oil and those with oil microcapsules were sufficient for the sausage producer to declare high content of these fatty acids in accordance with the current EC regulation.

## 1. Introduction

The worldwide consumer trend is to look for food that not only satisfies basic nutritional needs but is also beneficial for health and the function of the human body. Therefore, the market for functional food has developed rapidly in recent years. A food product can be classified as functional food if its ingredients come from natural sources and their consumption regulates the physiological functions of the human body [1]. The idea of producing food with an increased influence on health is based on enriching original products with health-promoting ingredients such as bioactive compounds and nutraceuticals [2].

Meat is a nutritious component of the diet. It is highly digestible, rich in proteins, essential amino acids, vitamins (especially B vitamins), and minerals (zinc and iron) [3]. Therefore, it is a good matrix for designing innovative products. Nowadays, consumers expect functional meat products with reduced content of salt, nitrates III and V, cholesterol, and fat [4]. Consumer demand for meat products containing functional ingredients is also growing steadily [5,6,7,8]. One of such ingredients is fish oil, whose beneficial effect on the human body has been proven in numerous studies. It is known as a rich source of long-chain n-3 polyunsaturated fatty acids, including docosahexaenoic acid (DHA; 22: 6n-3) and eicosapentaenoic acid (EPA; 20: 5n-3) [9]. These acids are credited with the prevention of cardiovascular diseases, colorectal, breast, and prostate cancers, as well as Alzheimer’s disease [9,10,11,12]. Apart from that, the fatty acids contained in fish oil are taken into consideration in the treatment of other diseases such as obesity, type 2 diabetes, depression, non-alcoholic fatty liver disease, and inflammations, and they are beneficial for the brain, memory, and reaction time [13,14,15]. Studies have shown that apart from reducing the risk of cardiovascular disease, n-3 fatty acids also improve the heart rate, reduce the risk of heart attack, regulate blood pressure, blood lipids, and arteriosclerosis [13].

The high content of n-3 acids in fish oil and their beneficial effect on human health contributed to it being recognised as a functional ingredient. The consumption of fish and seafood is a way of meeting the daily demand for n-3 fatty acids. It may be a good solution to consume products enriched with fish oil in order to prevent deficiency in n-3 fatty acids and reduce the risk of chronic diseases. One of the limitations of the use of fish oil in the food industry is the high susceptibility of n-3 fatty acids to oxidation, as well as changes in the flavour profile of the finished products. These processes can be limited by adding various antioxidants, other additives or spices to mask the fishy flavour or by using oil capsules [16]. Microencapsulation is a unique technique used to coat or encapsulate sensitive components as a core material in another wall material, which consists of a protective shell or wall. Microencapsulation helps to protect the core material from adverse environmental conditions (light, moisture, and oxygen). It also provides the possibility to control the release of ingredients at a specific time and place, mask the aftertaste or smell, modify physical characteristics by converting a liquid into a solid, and improve oxidative stability [17,18]. Microencapsulation limits the perceptibility of the fishy smell of oil, provided that the preparation does not dissolve immediately after being added to food [19]. Earlier studies showed that poultry meat could be successfully used as a raw material for products enriched with unsaturated fatty acids [20].

Thus far, research on meat fortification with n-3 acids has focused on determining the oxidative stability during storage and evaluating the sensory quality of finished meat products. There is much less information on the fatty acid profile and the bioavailability of microencapsulated n-3 fatty acids in finely-comminuted sausages. Therefore, the aim of this study was to produce, under commercial conditions, vacuum-packed (VP) and modified-atmosphere-packed (MAP) poultry sausages with liquid fish oil and microencapsulated fish oil additives and to determine their fatty acid profiles during storage. The oxidative stability of experimental sausages will be evaluated by way of periodical analysis for thiobarbituric acid reactive substances (TBARS) for storage time. Additionally, the bioavailability of n-3 acids in a microencapsulated fish oil additive during in vitro digestion of obtained products has also been studied.

## 2. Results and Discussion

### 2.1. Basic Analyses

The analysis of the basic chemical composition of the poultry sausages (Table 1) did not reveal any statistically significant differences between the control sample and the samples with the liquid (FO) and microencapsulated fish oil (MC) additives (*p* > 0.05). However, the sausages with the microcapsules had a slightly higher protein content (19.1%) than the other samples (18.7% on average). It is most likely that this effect was caused by the addition of oil to the microcapsules, whose matrix was porcine gelatine. Josquin et al. [21] and Stangierski et al. [22] observed a similar dependence in their studies. As far as the water and fat content is concerned, there was a similar dependence as in earlier studies [23,24]. The sausages with liquid oil had a slightly higher fat content (12.1%), whereas the lowest fat content was noted in the control samples (11.7%), which also had the highest water content (66.6%) (*p* > 0.05). There was also a lower water content in fresh and cooked pork burgers and Spanish salchichón with microencapsulated fish oil [25,26].

The results of an earlier study conducted on analogous samples of sausages included a detailed analysis of changes in the pH value and water activity (A_w_), depending on the form of fish oil additive, packaging method, and storage period [23]. Therefore, these results were not analysed in this study. The analyses conducted in the previous study showed a slight effect of the additive and the packaging method on the pH and water activity values. The initial pH value in all samples ranged from 5.83 to 5.96. On the 21st day of storage, the pH of the vacuum-packed sausage increased slightly to 6.15, whereas the pH of the MAP sample was 5.99. In the same period of time, the control samples and the MAP samples with microcapsules had the lowest pH, i.e., 5.80 on average.

The initial A_w_ values in all sausage variants were similar and amounted to 0.859 ± 0.001 on average. Neither the form of the oil additive nor the packaging method had a significant effect on the A_w_ value (*p* > 0.05). During storage, the A_w_ value in all the samples gradually decreased to a mean value of 0.852 ± 0.001.

### 2.2. Sensory Assessment

In general, the results of the sensory assessment obtained in this work do not allow for the division of the poultry sausages according to the packaging method because it did not have a significant influence on the results of the sensory evaluation, neither on the 1st nor the 21st day of storage (*p* > 0.05). In earlier studies by Stangierski et al. [22] and Kawecki et al. [24], which were performed as separate production batches with different sensorial panels, similar conclusions were formulated.

None of the differences observed in the sensory evaluation were statistically significant (*p* > 0.05). On the first day of storage, the sausage samples did not differ in the appearance of their external surface and cross-section. The sausages with the microencapsulated oil additive (MC) were rated the highest for colour (Table 2). The FO samples were slightly lighter in colour, and they had a slightly looser consistency than the other samples. On the 21st day of storage, all samples were rated slightly lower for their external appearance, surface colour, cross-sectional colour, taste, smell, and cold consistency than on the 1st day of storage. The only exception was the sausages with the liquid oil additive, as their cross-sectional colour and cold consistency were rated identically both at the beginning and at the end of the storage period. When the sausages were heated, there were no differences in their smell, regardless of the type of sample and storage time. The control samples (CO) were rated highest for their taste. At the same time, the sausages with the liquid oil additive did not differ in taste from the ones with oil microcapsules. The taste of individual samples did not differ significantly depending on the storage time. The MC and CO samples were rated highest for hot consistency, but after 21 days of storage, the latter samples were rated the same as those with the liquid oil additive. At the same time, the duration of storage did not affect the hot consistency of either type of fortified sausages.

Jiménez-Martín et al. [27] found that the microencapsulated fish oil additive had no effect on the organoleptic characteristics of chicken nuggets. However, the liquid fish oil additive positively affected the juiciness of the chicken nuggets and reduced the perceptibility of the characteristic meat aroma. Similarly, Solomando et al. [28] found that the microencapsulated fish oil additive did not cause any differences in the organoleptic evaluation of scalded poultry sausages. On the other hand, Raeisi et al. [29] conducted a similar study and found that the liquid fish oil additive significantly diminished all sensory discriminants of the chicken nuggets, as compared with the control sample and the samples with microencapsulated fish oil.

### 2.3. Fatty Acid Profile

Neither the liquid nor microencapsulated fish oil additive had a significant effect on the total fat content in the sausages, but it diversified the fatty acid profile (Table 3). Due to the raw materials used for the production of sausages, the following fatty acids were dominant: oleic acid, linoleic acid, and palmitic acid, which were followed by palmitoleic acid, stearic acid, myristic acid, lauric acid, and linolenic acid. Both forms of fish oil added to the meat stuffing enriched the fatty acid profile with eicosapentaenoic and docosahexaenoic acids. This observation is consistent with the findings of earlier studies [21,30].

The results of the multivariate analysis of variance (Table 4) showed that the fatty acid profile was significantly influenced by both the fish oil additive itself and its form. Only the amounts of palmitic, palmitoleic, and eicosadienoic acids were not affected by the type of sample. The storage conditions, or in fact the packaging method, significantly influenced the content of lauric, *cis*-oleic, eicosapentaenoic, and docosahexaenoic acids.

The storage time did not affect the proportion of only palmitic, α-linolenic, and eicosic acids in the fatty acid profile. The analysis of the interdependent effect of more than one factor on the fatty acid profile showed that the type of sample and packaging method, as well as the type of sample and storage time, significantly influenced the content of essential unsaturated fatty acids added with the oil, i.e., eicosapentaenoic acid and docosahexaenoic acid. The joint interaction of all three differentiating factors, i.e., the type of sample, packaging method, and storage time, was observed only for lauric and *cis*-oleic acids. The total content of eicosapentaenoic and docosahexaenoic acids expressed as g∙100 g^−1^ of the product changed significantly depending on the type of sample, packaging method, and storage time, or if the interaction of two factors is taken into account, the content of these acids changed significantly depending on the combined effect of the type of sample and packaging method as well as the combined effect of the type of sample and storage time.

The initial content of these acids in the sample with liquid fish oil was 0.22 g∙100 g^−1^ of the finished product. During 21-day storage, the content of these acids decreased to 0.18 g∙100 g^−1^ in the MAP sample and to 0.17 g∙100 g^−1^ in the VP sample.

### 2.4. Fatty Acid Profile in Sample Subjected to In Vitro Digestion

The gas chromatography analysis of the fatty acid profile of the sausages showed significant differences in the content of eicosapentaenoic and docosahexaenoic acids between the sample with liquid oil (FO) and the one with microencapsulated oil (MC) (*p* < 0.05). The content of these acids in the sample with oil microcapsules was nearly ten times lower than in the samples with liquid oil. It is likely that this effect was caused by the closure of a significant amount of fatty acids inside the capsule matrix, from which they were not released in a typical way. The method of encapsulating fat globules in the matrix was described in our previous study [22]. The release of these acids from the matrix of microcapsules (Table 5) was observed only when the sausage with the microencapsulated oil additive (MC) was subjected to simulated digestion (in vitro test). The model proposed in our study recreated basic digestion mechanisms occurring in a living organism under extracorporeal conditions, this enabled verification of the course of the digestion and assessment of the availability of nutrients.

Having passed through the ‘stomach’ stage, a significant amount of EPA and DHA was released at the initial section of the ‘small intestine’. The nature of changes in the in vitro digestion process was influenced by the pH value, the residence time, and the temperature of 37 °C. This was manifested by changes in the content of C:18 n-6 and n-3 acids, as well as C22:6 n-3 acid. The amount of these acids gradually decreased when they were released after the ‘stomach’ stage and the initial section of the ‘small intestine’. Solomando et al. [31,32] also observed that the greatest amount of EPA and DHA was released from microcapsules during digestion in the small intestine. These authors also found that the characteristics of microcapsules, as well as the system into which they were introduced, affected both the amount and bioavailability of EPA and DHA. They also observed that during in vitro digestion, the amount of EPA and DHA released from scalded sausages was greater than the amount released from dried ripened sausages. The amount of n-3 acids released from microencapsulated oil depends on the microencapsulation method, which affects the external and internal oil content in microcapsules [33]. Although research has been conducted for many years, the mechanisms involved in lipid digestion in complex systems have not been fully explained. The release of significant amounts of EPA and DHA during digestion in the small intestine, followed by a gradual decrease in the DHA content and an increase in the EPA content in the subsequent phases of the digestion process, may be influenced by the physicochemical properties of fat droplets in emulsions, such as their size, the molecular structure of triacylglycerols and the surface organisation and composition [34].

After a 21-day storage period, the total EPA and DHA content in the sausages with the liquid fish oil additive amounted to 0.18 g∙100 g^−1^ of the product in the MAP sample and 0.17 g∙100 g^−1^ of the product in the VP sample. After in vitro digestion in the ‘stomach’ and in the initial section of the ‘small intestine’, the total EPA and DHA content in the sample with microencapsulated oil amounted to 0.17 g∙100 g^−1^ of the product. This indicates that in our experiment, the content of these acids in the model poultry products fortified with oil and microcapsules was stable during storage. In consequence, the producer can declare that sausages produced in this way have a high content of n-3 acids, in accordance with the European Commission Regulation (EU) No. 116/2010.

### 2.5. TBARS Analysis

**Figure 1:**
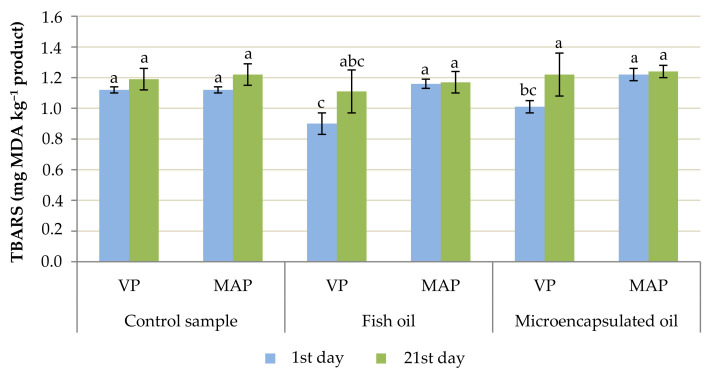
The effect of the liquid and microencapsulated fish oil additive, packaging method and storage time on the oxidative stability of the poultry sausages. a–c = The mean values with the same superscript are not significantly different (*p* < 0.05; mean ± standard deviation; *n* = 6).

Figure 1 shows how the fish oil added to the poultry sausages affected the TBARS value measured on the 1st and 21st days of refrigerated storage.

The lowest values were noted on the first day in the vacuum-packed samples with both forms of the oil additive, i.e., 0.90 ± 0.07 for the FO sample and 1.01 ± 0.04 for the MC sample (TBARS; mg MDA kg^−1^ product). There were no significant differences (*p* > 0.05) observed in the other samples. The storage of the samples for 21 days slightly increased the oxidation of lipids in all sausages. The mean TBARS value in the VP samples was 1.17 mg MDA kg^−1^ product, whereas in the MAP samples, it was 1.21 mg MDA kg^−1^ product. On the 21st day of storage, the TBARS values in the VP samples were slightly lower than in the MAP samples, but the difference was not significant (*p* > 0.05). These results differ from the observations made by Dominguez et al. [35], who replaced part of the pork fat in frankfurters with microencapsulated fish oil. They noted the highest TBARS value in the samples with the oil microcapsules. On the other hand, Josquin et al. [21] conducted an experiment on Dutch-style fermented sausages and found the lowest TBARS value in the samples with the microencapsulated fish oil additive. In their opinion, this result stemmed from the fact that the capsule protected unsaturated fatty acids from oxidation. In our study, at the end of the storage period, there were no differences in the TBARS value between individual samples. This may have been caused by the protective effect of the microcapsule, the additives (such as sodium ascorbate), and appropriate packaging methods, which effectively protected the product from oxidative changes during storage.

## 3. Materials and Methods

The aim of the study was to produce poultry sausages enriched with n-3 acids in a sufficient amount, to declare the high content of these fatty acids in accordance with European Commission Regulation (EU) No. 116/2010. According to this regulation, in order to declare a high content of n-3 fatty acids in a product, it must contain at least 80 mg of eicosapentaenoic acid (EPA) and docosahexaenoic acid (DHA), in total per 100 g of the product and 100 kcal of the product.

### 3.1. Sample Preparation

The experimental sausages were produced industrially from the following raw materials: chicken breast (60%), chicken leg meat (22%), and chicken skin (18%). A standard set of additives was used, i.e., salt (1.4%), sodium ascorbate (0.1%), spices and flavourings (3.2%), and water (20%). The meat was comminuted in a grinder with a 5 mm mesh, and then the other ingredients were added and mixed thoroughly. The stuffing was divided into three equal portions: a control sample (CO), a sample with liquid fish oil added (FO) and a sample with microencapsulated fish oil (ME). The final temperature of the stuffing was 8–10 °C. The stuffing was put into cellulose casings with a diameter of 13 mm. Next, standard technological procedures were followed, i.e., settling, drying, smoking (60 °C), steaming (75 °C), and cooling. The average production process efficiency was 86%. The resulting sausages were packed in a vacuum or in a modified atmosphere, with five pieces in each package, and stored at 4 ± 2 °C. The gas mixture was composed of: 0.06% O_2_, 72.0% N_2_, and 27.9% CO_2_.

The following oil preparations were used (DSM Nutritional Products Ltd., Basel, Switzerland): 30% 8a MEG-3™ cooking oil consisting of refined fish oil and fish oil microencapsulated in 30% MEG-3™ porcine gelatine powder. According to the manufacturer’s certificate, the 30% 8a MEG-3™ cooking oil contained n-3 polyunsaturated fatty acids (PUFA) such as triglycerides (TG), including eicosapentaenoic acid (EPA) and docosahexaenoic acid (DHA). The total content of n-3 fatty acids was at least 300 mg∙g^−1^. The total EPA and DHA content in the preparation was 25%. The 30% MEG-3™ powder contained at least 60% fish oil, 30% of which consisted of total n-3 polyunsaturated fatty acids (PUFA) such as triglycerides, including eicosapentaenoic acid (EPA) and docosahexaenoic acid (DHA), microencapsulated in porcine gelatine. The total EPA and DHA content in the preparation was 15%. The data on the raw material composition and the manufacturer’s declaration regarding the EPA + DHA content in the oil preparations were used to determine the amount of 30% 8a MEG-3™ cooking oil and 30% MEG-3™ powder added to the stuffing at 7.1 g∙kg^−1^ and 11.9 g∙kg^−1^, respectively. As a result, the EPA and DHA content in the finished product was 2.2 g∙kg^−1^.

### 3.2. Basic Chemical Composition

The basic chemical composition of the sausages, i.e., the content of water, protein (the conversion factor value was 6.25), fat, and ash, was determined using the methods approved by the Polish Committee for Standardisation [36,37,38,39]. The basic composition was tested on the 1st day of storage of the sausages.

### 3.3. Sensory Assessment

The sausages were assessed by a ten-member trained sensory panel in accordance with the requirements of the PN-EN ISO 8586:2014–03 standard [40]. The following sensory characteristics were assessed: external appearance, external colour, cross-sectional appearance, cross-sectional colour, smell, taste, and consistency of the cold and heated product. The individual sensory characteristics were rated on a five-point scale with the following degrees of intensity: very attractive, distinctive, standard (5 points), attractive, with small deviations (4 points), average, with noticeable deviations (3 points), inadequate, with significant deviations (2 points), unacceptable (1 point). The scaling method [41] was used to quantify the intensity of selected sensory characteristics. The sausages were assessed on the 1st and 21st days of cold storage.

### 3.4. Fatty Acid Analysis

The modified method described by Folch et al. was used to extract fat from the poultry sausages [42]. A 5 g sample (± 0.01 g) was shaken for about 18 h on an Incu-Shaker Mini (Syreville, NY, USA) laboratory shaker with a 100 mL mixture of chloroform and methanol (2:1; v:v). The extracted lipids were filtered through filter paper and mixed with a 0.88% sodium chloride solution. After the separation phase, the upper layer was removed completely and then the chloroform layer was carefully evaporated under nitrogen. Next, each sample was saponified (20 min, 60 °C) with 0.5 M KOH in methanol. Then the free fatty acids were methylated with a 14% (v:v) mixture of BF_3_-MeOH (15 min, 60 °C). Finally, fatty acid methyl esters (FAME) were extracted with hexane. The fatty acid profile of the experimental sausages was analysed with a QP 5050A gas chromatography analyser (Shimadzu, Duisburg, Germany). The FAME mixtures were analysed with a gas chromatograph equipped with a SP™—2560 silica capillary column (100 × 25 mm; layer thickness 25 µm; Supelco Inc., Bellefonte, PA, USA) and a flame ionisation detector. The carrier gas was helium, with a flow rate of 1.8 mL min^−1^. The injector and detector temperatures were set at 245 °C with an injection volume of 1 µL. The initial oven temperature of 60 °C was maintained for 5 min and then gradually increased by 15 °C min^−1^ up to 180 °C. It was kept at this level for 16 min, and then it was gradually increased by 5 °C min^−1^ to 220 °C and maintained for 7 min. The entire analytical programme lasted 60 min. The fatty acid methyl ester profile was expressed as g∙100 g^−1^ total fatty acids. The fatty acid profile in the sausages was analysed on the 1st and 21st days of storage.

### 3.5. In Vitro Digestion Conditions

**Scheme 1:**
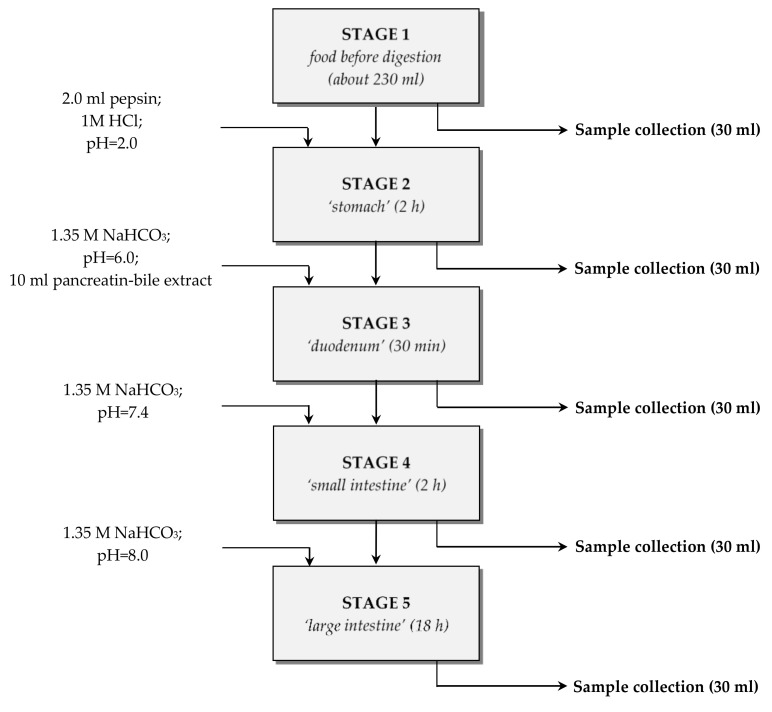
A schematic diagram of the in vitro digestion process in a gastrointestinal tract model.

The in vitro digestion test of the sausage with the microencapsulated oil additive was conducted on the 21st day of vacuum storage. A sample of 20 ± 0.001 g of sausage underwent simulated chewing with 200 mL of deionised water in a stomacher for 1 min. Next, it underwent in vitro digestion, according to the scheme shown below (Scheme 1).

The reaction tank was a glass vessel with a glass lid with 4 inlets enabling: the insertion of a pH electrode, programming of active acidity with a peristaltic pump with the possibility of recording changes in active acidity, the dosage of biochemical agents, and collection of samples for analysis. The reaction tank was thermostatted. The temperature of 37 °C was maintained by means of a water bath. A magnetic stirrer ensured the homogeneity of the reaction conditions. The conditions of the ‘digestion’ process in the bioreactor were prepared according to the method described by Hoebler et al. [43] and our research. The digestion process consisted of the following stages: ‘stomach’, ‘small intestine’, and ‘large intestine’ [44]. The entire ‘digestion’ process lasted 24 h. The following enzymes and extracts were used in the digestion process:

- pepsin from porcine gastric mucosa (P7000, Sigma-Aldrich, St Louis, MO, USA);
- pancreatin from porcine pancreas (P1750, Sigma-Aldrich, St Louis, MO, USA);
- bovine bile (Sigma B-8381, Sigma-Aldrich, St Louis, MO, USA).

### 3.6. TBARS Analysis

Lipid oxidation was assessed with 2-thiobarbituric acid (TBA) according to the method invented by Salih et al. [45]. Pieces weighing 10 ± 0.001 g were taken from each of the sausage samples. They were homogenised with 30.0 mL of a cold (4 °C) extraction solution containing 4% perchloric acid and 0.75 mL of butylated hydroxyanisole. The mixed samples were filtered and diluted with 50 mL of 4% perchloric acid. After mixing 5.0 mL of the filtrate, it was added to 5.0 mL of 0.02 M TBA. The test tubes were sealed and heated in a water bath for 60 min at 100 °C and then cooled. The absorbance was measured with a UV-1800 spectrophotometer (Shimadzu Corporation, Kyoto, Japan) at a wavelength of 532 nm against a blank containing 5.0 mL of 4% perchloric acid and 5.0 mL of 0.02 M TBA solution. The results were expressed as TBARS in mg of malondialdehyde (MA) kg^−1^ of the sample, using a standard curve prepared from 1,1,3,3-tetramethoxypropane according to the formula: TBARS = AK (mg MA∙kg^−1^), where: A = absorbance of the sample and K = a conversion factor of 5.5. The TBARS measurements were made on the 1st and 21st days of cold storage of the sausages.

### 3.7. Statistical Analyses

The significance of differences between mean values within one discriminant was calculated by means of Duncan’s test. The significance level was *p* < 0.05. Multivariate ANOVA was used to detect the effects of interaction between selected variables and their effect on the fatty acid profile. Statistica 13.1 software (StatSoft Inc. Tulsa, OK, USA) was used for statistical analyses. The experiments were conducted twice (two different production batches), and all the tests were replicated at least three times.

## 4. Conclusions

The results of the study led to the conclusion that neither the liquid nor microencapsulated fish oil additive had a significant influence on the basic composition of the sausages or the results of their sensory evaluation. All the samples were similarly rated by the evaluation panel. The liquid oil additive did not leave a negative fishy aftertaste. It may have been caused by the fact that refined oil was used in our experiment, as well as protective and seasoning additives.

The results of the TBARS analysis showed that the oil additive did not affect the degree of lipid oxidation. There were no statistically significant differences in the values of this index after 21 days of storage of the sausages, regardless of the form of the oil additive. Both forms of fish oil were added to the sausages in sufficient amounts not only to meet but also to exceed the required minimum EPA and DHA content in the finished product, as specified by the European Commission Regulation (EU) No. 116/2010. This means that the producer can declare a high content of n-3 acids in the finished products. However, when microcapsules are used, it is difficult to specify the profile of the fatty acids contained in the oil. Gas chromatography analysis of the profile of fatty acids contained in the sausages with the microencapsulated oil additive did not reveal the presence of EPA and DHA in the samples. Their presence was noted only when the in vitro digestion of the sausages was simulated. It is extremely important when controlling the content of these acids added to products in microcapsules. In order to protect EPA and DHA from changes during the storage of the product, the MAP method seems to be more effective than vacuum-packing.

In view of the aforementioned fact, liquid, rather than microencapsulated fish oil, should be added to poultry sausages to enrich them with n-3 acids. This is justified both in terms of cost-effectiveness and production because liquid oil is cheaper than microencapsulated oil, and it is much more difficult to evenly distribute microencapsulated oil in the stuffing. It is easier to use liquid oil in industrial production. If food inspectors want to evaluate the product, they can easily determine its actual EPA and DHA content.

## Figures and Tables

**Table 1:** The basic chemical composition of the poultry sausages.

Chemical		Type of Sample	
Composition (%)	Control Sample (CO)	Fish Oil (FO)	Microencapsulated Oil (MC)
Moisture	67.2 ± 0.6	66.9 ± 0.4	66.6 ± 0.6
Protein	18.8 ± 0.4	18.6 ± 0.3	19.1 ± 0.4
Fat	11.5 ± 0.3	12.1 ± 0.2	11.7 ± 0.3
Ash	2.4 ± 0.0	2.3 ± 0.0	2.4 ± 0.0

No statistically significant differences between the mean values in the same rows (*p* < 0.05; mean ± standard deviation; *n* = 6).

**Table 2:** The intensity of the sensory characteristics of the model systems of poultry sausages on the 1st and 21st days of storage.

Sensory Characteristics			Type of Sample			
	Control Sample (CO)		Fish Oil (FO)		Microencapsulated Oil (MC)	
	1st Day	21st Day	1st Day	21st Day	1st Day	21st Day
External appearance	4.1 ± 0.4	4.0 ± 0.4	4.1 ± 0.3	3.9 ± 0.4	4.1 ± 0.3	4.0 ± 0.3
External colour	4.1 ± 0.3	4.0 ± 0.3	4.1 ± 0.4	4.0 ± 0.4	4.2 ± 0.3	4.0 ± 0.3
Cross-sectional appearance	4.2 ± 0.3	4.2 ± 0.3	4.2 ± 0.3	4.2 ± 0.3	4.2 ± 0.3	4.3 ± 0.3
Cross-sectional colour	4.2 ± 0.4	4.1 ± 0.4	4.1 ± 0.3	4.1 ± 0.3	4.3 ± 0.3	4.1 ± 0.3
Smell of cold product	4.3 ± 0.3	4.2 ± 0.3	4.2 ± 0.3	4.1 ± 0.3	4.3 ± 0.3	4.1 ± 0.3
Taste of cold product	4.3 ± 0.4	4.1 ± 0.3	4.2 ± 0.4	4.0 ± 0.4	4.2 ± 0.4	4.0 ± 0.4
Consistency of cold product	4.5 ± 0.2	4.4 ± 0.3	4.4 ± 0.3	4.4 ± 0.3	4.5 ± 0.2	4.4 ± 0.3
Smell of heated product	4.2 ± 0.4	4.2 ± 0.4	4.2 ± 0.4	4.2 ± 0.4	4.2 ± 0.4	4.2 ± 0.4
Taste of heated product	4.3 ± 0.3	4.3 ± 0.3	4.2 ± 0.4	4.2 ± 0.4	4.2 ± 0.3	4.2 ± 0.3
Consistency of heated product	4.5 ± 0.2	4.4 ± 0.2	4.4 ± 0.3	4.4 ± 0.3	4.5 ± 0.2	4.5 ± 0.2

No statistically significant differences between the mean values in the same rows (*p* < 0.05; mean ± standard deviation; *n* = 20).

**Table 3:** The profile of fatty acids in the poultry sausages with the liquid and microencapsulated fish oil additive depending on the packaging method (vacuum-packed-VP or modified-atmosphere-packed-MAP) and storage duration.

Fatty Acids	Control Sample (CO)				Fish Oil (FO)				Microencapsulated (MC)			
	MAP		VP		MAP		VP		MAP		VP	
	1st Day	21st Day	1st Day	21st Day	1st Day	21st Day	1st Day	21st Day	1st Day	21st Day	1st Day	21st Day
C12:0 (lauric acid)	2.40 ± 0.06 ^a^	2.01 ± 0.08 ^a^	2.03 ± 0.12 ^a^	1.85 ± 0.08 ^b^	2.00 ± 0.05 ^a^	1.83 ± 0.04 ^b^	2.08 ± 0.05 ^a^	1.74 ± 0.04 ^cb^	2.15 ± 0.13 ^a^	1.84 ± 0.09 ^b^	2.30 ± 0.03 ^a^	1.82 ± 0.13 ^b^
C14:0 (myristic acid)	1.98 ± 0.06 ^a^	2.02 ± 0.10 ^ad^	2.03 ± 0.03 ^ad^	1.97 ± 0.10 ^a^	2.40 ± 0.02 ^b^	2.23 ± 0.07 ^c^	2.40 ± 0.03 ^b^	2.19 ± 0.08 ^c^	2.12 ± 0.02 ^cd^	1.95 ± 0.11 ^a^	2.01 ± 0.05 ^a^	1.93 ± 0.15 ^a^
C15:0 (pentadecanoic acid)	0.08 ± 0.01 ^a^	0.09 ± 0.01 ^be^	0.08 ± 0.01 ^ae^	0.08 ± 0.01 ^ae^	0.13 ± 0.01 ^c^	0.14 ± 0.01 ^d^	0.13 ± 0.01 ^c^	0.14 ± 0.00 ^cd^	0.09 ± 0.01 ^ef^	0.10 ± 0.00 ^bf^	0.10 ± 0.01 ^bf^	0.09 ± 0.01 ^ae^
C16:0 (palmitic acid)	19.90 ± 0.07 ^a^	20.26 ± 1.56 ^a^	20.05 ± 0.20 ^a^	20.30 ± 1.59 ^a^	20.17 ± 0.20 ^a^	19.29 ± 1.04 ^a^	20.05 ± 0.14 ^a^	19.50 ± 1.17 ^a^	20.74 ± 0.31 ^a^	19.87 ± 1.21 ^a^	19.62 ± 0.52 ^a^	19.64 ± 1.82 ^a^
C16:1n-7 (palmitolenic acid)	5.77 ± 0.06 ^a^	5.95 ± 0.36 ^ac^	5.74 ± 0.11 ^a^	5.94 ± 0.49 ^ad^	5.65 ± 0.13 ^a^	6.38 ± 0.48 ^bcde^	5.72 ± 0.03 ^a^	5.93 ± 0.25 ^a^	5.68 ± 0.11 ^a^	5.82 ± 0.41 ^a^	5.64 ± 0.06 ^a^	6.10 ± 0.76 ^ae^
C18:0 (stearic acid)	5.07 ± 0.11 ^a^	3.91 ± 0.25 ^b^	4.91 ± 0.07 ^a^	3.59 ± 0.30 ^bc^	3.65 ± 0.03 ^bd^	3.24 ± 0.41 ^c^	3.60 ± 0.10 ^bc^	3.51 ± 0.13 ^a^	3.80 ± 0.21 ^b^	3.36 ± 0.17 ^cd^	3.30 ± 0.27 ^cd^	3.32 ± 0.64 ^cd^
C18:1n-9 + n-7 (*cis*-oleic acid)	37.65 ± 0.35 ^adf^	38.08 ± 0.38 ^adf^	37.39 ± 0.14 ^acf^	38.84 ± 0.50 ^b^	36.47 ± 0.04 ^ce^	37.44 ± 0.28 ^df^	36.52 ± 0.32 ^a^	37.82 ± 0.27 ^ad^	37.32 ± 0.11 ^f^	38.65 ± 0.29 ^bg^	38.44 ± 0.25 ^bg^	38.76 ± 0.83 ^bg^
C18:2n-6 (linoleic acid)	23.44 ± 0.27 ^ac^	24.16 ± 0.91 ^a^	23.89 ± 0.02 ^a^	23.98 ± 0.94 ^a^	22.42 ± 0.17 ^b^	23.18 ± 0.74 ^ab^	22.62 ± 0.18 ^b^	23.17 ± 0.86 ^ab^	23.38 ± 0.11 ^ab^	23.98 ± 0.90 ^a^	24.11 ± 0.31 ^a^	24.13 ± 1.22 ^a^
C18:3n-3 (α-linolenic acid)	1.41 ± 0.01 ^a^	1.39 ± 0.15 ^a^	1.43 ± 0.04 ^a^	1.40 ± 0.15 ^a^	1.40 ± 0.05 ^b^	1.39 ± 0.10 ^a^	1.42 ± 0.05 ^b^	1.45 ± 0.12 ^ad^	1.31 ± 0.10 ^bc^	1.34 ± 0.13 ^bcd^	1.28 ± 0.03 ^bc^	1.33 ± 0.02 ^abd^
C18:3n-6 (ɣ-linolenic acid)	nd	nd	nd	nd	0.19 ± 0.02 ^a^	0.23 ± 0.05 ^b^	0.23 ± 0.03 ^b^	0.21 ± 0.04 ^ab^	0.21 ± 0.02 ^ab^	0.10 ± 0.02 ^c^	0.20 ± 0.03 ^ab^	0.06 ± 0.03 ^d^
C20:1 (eicosenoic acid)	0.37 ± 0.04 ^ab^	0.34 ± 0.04 ^a^	0.37 ± 0.03 ^ab^	0.46 ± 0.01 ^b^	0.57 ± 0.03 ^c^	0.57 ± 0.01 ^c^	0.57 ± 0.01 ^c^	0.59 ± 0.02 ^c^	0.36 ± 0.09 ^ab^	0.39 ± 0.19^ab^	0.37 ± 0.02 ^ab^	0.41 ± 0.03 ^ab^
C20:2n-6 (eicosadienic acid)	0.25 ± 0.01 ^a^	0.18 ± 0.03 ^b^	0.26 ± 0.03 ^a^	0.20 ± 0.01 ^bc^	0.22 ± 0.02 ^cd^	0.20 ± 0.01 ^be^	0.23 ± 0.02 ^ad^	0.19 ± 0.01 ^be^	0.24 ± 0.03 ^ad^	0.21 ± 0.01 ^bc^	0.22 ± 0.03 ^cde^	0.20 ± 0.01 ^bc^
C20:4n-6 (arachidonic acid)	0.77 ± 0.12 ^ac^	0.51 ± 0.12 ^b^	0.78 ± 0.05 ^ac^	0.45 ± 0.02 ^b^	0.82 ± 0.01 ^a^	0.54 ± 0.02 ^b^	0.82 ± 0.02 ^a^	0.50 ± 0.01 ^b^	0.72 ± 0.01 ^c^	0.48 ± 0.03 ^b^	0.70 ± 0.12 ^c^	0.49 ± 0.01 ^b^
C20:5n-3 (eicosapentaenoic acid; EPA)	nd	nd	nd	nd	0.76 ± 0.01 ^a^	0.66 ± 0.02 ^b^	0.73 ± 0.03 ^c^	0.63 ± 0.01 ^d^	0.08 ± 0.01 ^e^	0.06 ± 0.01 ^f^	0.09 ± 0.01 ^e^	0.05 ± 0.01 ^f^
C22:6n-3 (docosahexaenoic acid; DHA)	nd	nd	nd	nd	1.07 ± 0.06 ^a^	0.86 ± 0.05 ^b^	1.09 ± 0.13 ^a^	0.81 ± 0.01 ^c^	0.18 ± 0.01 ^d^	0.13 ± 0.02 ^de^	0.17 ± 0.02 ^d^	0.09 ± 0.01 ^e^
Σ EPA and DHA	-	-	-	-	1.82 ± 0.06 ^a^	1.52 ± 0.06 ^b^	1.82 ± 0.16 ^a^	1.44 ± 0.01 ^c^	0.26 ± 0.02 ^d^	0.19 ± 0.03 ^de^	0.25 ± 0.02 ^d^	0.13 ± 0.02 ^e^
Σ EPA and DHA (g∙100 g^−1^ product)	-	-	-	-	0.22 ± 0.01 ^a^	0.18 ± 0.01 ^b^	0.22 ± 0.02 ^a^	0.17 ± 0.00 ^c^	0.03 ± 0.00 ^d^	0.02 ± 0.00 ^d^	0.03 ± 0.00 ^d^	0.02 ± 0.00 ^d^
EPA (g∙100 g^−1^ product)	-	-	-	-	0.09 ± 0.00 ^a^	0.08 ± 0.00 ^b^	0.09 ± 0.00 ^a^	0.08 ± 0.00 ^c^	0.01 ± 0.00 ^d^	0.01 ± 0.00^de^	0.01 ± 0.00 ^d^	0.01 ± 0.00 ^e^
DHA (g∙100 g^−1^ product)	-	-	-	-	0.13 ± 0.01 ^a^	0.10 ± 0.01 ^b^	0.13 ± 0.01 ^a^	0.09 ± 0.00 ^c^	0.02 ± 0.00^d^	0.01 ± 0.00 ^de^	0.02 ± 0.00^d^	0.01 ± 0.00 ^e^

a–g = The mean values with the same superscript in the same row are not significantly different (*p* < 0.05; mean ± standard deviation; *n* = 6; nd = not detected).

**Table 4:** The effects of interactions between selected variables (type of sample, packaging method, storage time) and their influence on the fatty acid profile.

Fatty Acids	Type of Sample	Packaging Method	Storage Time (day)	Type of Sample × Packaging Method	Type of Sample × Storage Time (day)	Packaging Method × Storage Time (day)	Type of Sample × Packaging Method × Storage Time (day)
C12:0	0.035 *	0.020	<0.001	0.265	0.007	0.097	0.020
C14:0	<0.001	0.136	<0.001	0.459	0.003	0.667	0.148
C15:0	<0.001	0.278	0.018	0.355	0.007	0.018	0.369
C16:0	0.518	0.510	0.299	0.418	0.278	0.487	0.749
C16:1n-7	0.625	0.742	0.001	0.377	0.458	0.741	0.170
C18:0	<0.001	0.507	<0.001	0.451	0.359	0.050	0.168
C18:1n-9 + n-7	<0.001	<0.001	<0.001	0.164	0.411	0.549	<0.001
C18:2n-6	<0.001	0.213	0.012	0.678	0.712	0.184	0.866
C18:3n-3	0.002	0.658	0.733	0.687	0.542	0.665	0.906
C18:3n-6	<0.001	0.609	<0.001	0.036	<0.001	0.022	0.212
C20:1	<0.001	0.102	0.138	0.425	0.876	0.187	0.451
C20:2n-6	0.158	0.965	<0.001	0.184	0.010	0.895	0.261
C20:4n-6	0.003	0.415	<0.001	0.827	0.131	0.415	0.451
C20:5n-3	<0.001	0.006	<0.001	0.014	<0.001	0.309	0.468
C22:6n-3	0.002	0.012	<0.001	0.003	<0.001	0.098	0.735
Σ EPA and DHA	<0.001	0.102	<0.001	0.479	<0.001	0.122	0.495
Σ EPA and DHA (g∙100 g^−1^ product)	<0.001	0.006	<0.001	0.014	<0.001	0.309	0.468

* *p*-value (the significance level *p* < 0.05).

**Table 5:** The fatty acid profile of the sausage with the microencapsulated oil additive subjected to in vitro digestion.

Fatty Acids	Before Digestion	After Stomach Stage	pH 2.0–7.4 (Initial Section of Small Intestine)	2 h in Small Intestine	pH 7.4–8.0 (Initial Section of Large Intestine)	18 h pH 8.0 Large Intestine
C12:0	2.11 ± 0.24 ^a^	1.71 ± 0.18 ^b^	1.78 ± 0.15 ^b^	1.47 ± 0.20 ^c^	1.55 ± 0.11 ^c^	1.09 ± 0.09 ^d^
C14:0	1.51 ± 0.08 ^a^	1.21 ± 0.12 ^b^	2.81 ± 0.31 ^c^	2.88 ± 0.21 ^c^	2.72 ± 0.19 ^c^	2.03 ± 0.14 ^d^
C15:0	0.32 ± 0.09 ^a^	0.24 ± 0.07 ^a^	0.28 ± 0.07 ^a^	0.25 ± 0.05 ^a^	0.21 ± 0.08 ^a^	0.17 ± 0.07 ^a^
C16:0	23.60 ± 1.34 ^a^	21.39 ±1.27 ^b^	26.35 ± 1.41 ^c^	27.93 ± 1.38 ^d^	25.49 ± 1.51 ^c^	27.75 ± 1.39 ^d^
C16:1n-7	3.61 ± 0.23 ^a^	3.25 ± 0.29 ^b^	3.81 ± 0.19 ^a^	3.13 ± 0.22 ^b^	2.46 ± 0.16 ^c^	2.25 ± 0.23 ^c^
C18:0	3.53 ± 0.22 ^a^	2.93 ± 0.17 ^b^	4.29 ± 0.37 ^c^	5.62 ± 0.48 ^d^	4.82 ± 0.41 ^e^	5.14 ± 0.45 ^d^
C18:1n-9 + n-7	35.34 ± 2.15 ^a^	38.13 ± 2.34 ^b^	31.99 ± 2.29 ^c^	31.90 ± 2.53 ^c^	33.04 ± 1.99 ^c^	33.35 ± 2.19 ^c^
C18:2n-6	27.39 ± 1.64 ^a^	29.06 ± 1.51 ^b^	24.79 ± 1.42 ^c^	23.53 ± 1.41 ^c^	26.80 ± 1.37 ^c^	25.68 ± 1.44 ^c^
C18:3n-3	0.13 ± 0.03 ^a^	0.10 ± 0.03 ^a^	0.26 ± 0.07 ^b^	0.17 ± 0.05 ^c^	0.08 ± 0.01 ^a^	0.13 ± 0.09 ^a^
C18:3n-6	0.10 ± 0.02 ^a^	0.06 ± 0.03 ^b^	0.35 ± 0.09 ^c^	0.15 ± 0.06 ^a^	0.09 ± 0.02 ^ab^	0.10 ± 0.05 ^ab^
C20:1	2.11 ± 0.17 ^a^	1.74 ± 0.12 ^b^	1.33 ± 0.09 ^c^	1.33 ± 0.11 ^c^	1.20 ± 0.10 ^d^	1.00 ± 0.10 ^d^
C20:2n-6	0.08 ± 0.02 ^a^	0.09 ± 0.02 ^a^	0.32 ± 0.11 ^b^	0.35 ± 0.12 ^b^	0.27 ± 0.11 ^b^	0.28 ± 0.14 ^b^
C20:4n-6	0.04 ± 0.00 ^a^	0.04 ± 0.01 ^a^	0.03 ± 0.01 ^a^	0.16 ± 0.09 ^b^	0.18 ± 0.08 ^b^	0.15 ± 0.10 ^b^
C20:5n-3	0.13 ± 0.09 ^a^	0.05 ± 0.01 ^b^	0.35 ± 0.12 ^c^	0.38 ± 0.10 ^c^	0.50 ± 0.13 ^d^	0.48 ± 0.15 ^d^
C22:6n-3	nd	nd	1.26 ± 0.15 ^a^	0.75 ± 0.09 ^b^	0.59 ± 0.12 ^c^	0.40 ± 0.11 ^d^
Σ EPA and DHA	0.13 ± 0.05 ^a^	0.05 ± 0.01 ^b^	1.61 ± 0.21 ^c^	1.13 ± 0.19 ^d^	1.09 ± 0.14 ^d^	0.88 ± 0.12 ^e^
Σ EPA and DHA (g∙100 g^−1^ product)	0.01 ± 0.01 ^a^	0.01 ± 0.00 ^a^	0.17 ± 0.04 ^b^	0.12 ± 0.03 ^c^	0.11 ± 0.04 ^c^	0.09 ± 0.03 ^c^

a–e = The mean values with the same superscript in the same row are not significantly different (*p* < 0.05; mean ± standard deviation; *n* = 6; nd = not detected).

## Data Availability

All data is contained within the article.

## Sample Availability

Samples of the compounds are available from the authors.
